# Transiently Transfected Purine Biosynthetic Enzymes Form Stress Bodies

**DOI:** 10.1371/journal.pone.0056203

**Published:** 2013-02-06

**Authors:** Alice Zhao, Mark Tsechansky, Jagannath Swaminathan, Lindsey Cook, Andrew D. Ellington, Edward M. Marcotte

**Affiliations:** Center for Systems and Synthetic Biology, Institute for Cellular and Molecular Biology, University of Texas at Austin, Austin, Texas, United States of America; Semmelweis University, Hungary

## Abstract

It has been hypothesized that components of enzymatic pathways might organize into intracellular assemblies to improve their catalytic efficiency or lead to coordinate regulation. Accordingly, *de novo* purine biosynthesis enzymes may form a purinosome in the absence of purines, and a punctate intracellular body has been identified as the purinosome. We investigated the mechanism by which human *de novo* purine biosynthetic enzymes might be organized into purinosomes, especially under differing cellular conditions. Irregardless of the activity of bodies formed by endogenous enzymes, we demonstrate that intracellular bodies formed by transiently transfected, fluorescently tagged human purine biosynthesis proteins are best explained as protein aggregation.

## Introduction

Enzymes have previously been found to organize into intracellular assemblies that may improve their catalytic efficiency or lead to coordinate regulation. For example, the trypanosome glycolytic pathway is organized into a glycosome. Similarly, many such cellular bodies occur naturally, functioning in degradation or storage (e.g., P bodies [1] or actin bodies [2]).

While such functional bodies continue to be found, it is also the case that overexpression of proteins in cells can lead to aggregation [3], [4], such as the formation of inclusion bodies. While measuring the localization of green fluorescent protein (GFP)-tagged proteins, we identified a surprisingly large number of punctate bodies that accumulated in yeast cells during nutrient starvation [5]. This led us to further speculate whether such bodies were representative of endogenous, functional assemblies, or accidental or pathological aggregates.

Among >100 proteins forming such bodies, we observed that the *de novo* purine biosynthetic enzyme phosphoribosyl pyrophosphate amidotransferase (encoded by the yeast gene ADE4, homolog of the human enzyme PPAT) reversibly formed intracellular bodies in the presence and absence of adenine. We initially thought that such bodies might be depots for functional enzymes, and the observation of intracellular bodies associated both with the yeast Ade4 and the human PPAT enzyme suggested the possibility of a functional purine-biosynthetic intracellular body conserved between yeast and humans. In particular, Benkovic and co-workers have identified a cellular body (composed in part of the PPAT enzyme) that they called the purinosome [6], forming in human cell culture in the absence of purines, whose assembly has been shown to be assisted by microtubules and perturbed by casein kinase II inhibitors [7], [8] and which may be under GPCR control [9].

However, the possibility remained that the manipulation of the gene (via fusion to GFP) or its expression (via transfection and / or starvation) [6] had led to aggregate formation. Therefore, we sought to establish whether the observed punctate bodies [6] were formed by transiently expressing the recombinant enzymes in cultured cells. We have now characterized punctate formation in greater depth and devised explicit tests to determine whether the bodies may have arisen due to non-native protein expression, stress, or aggregation.

## Results and Discussion

**Figure 1:**
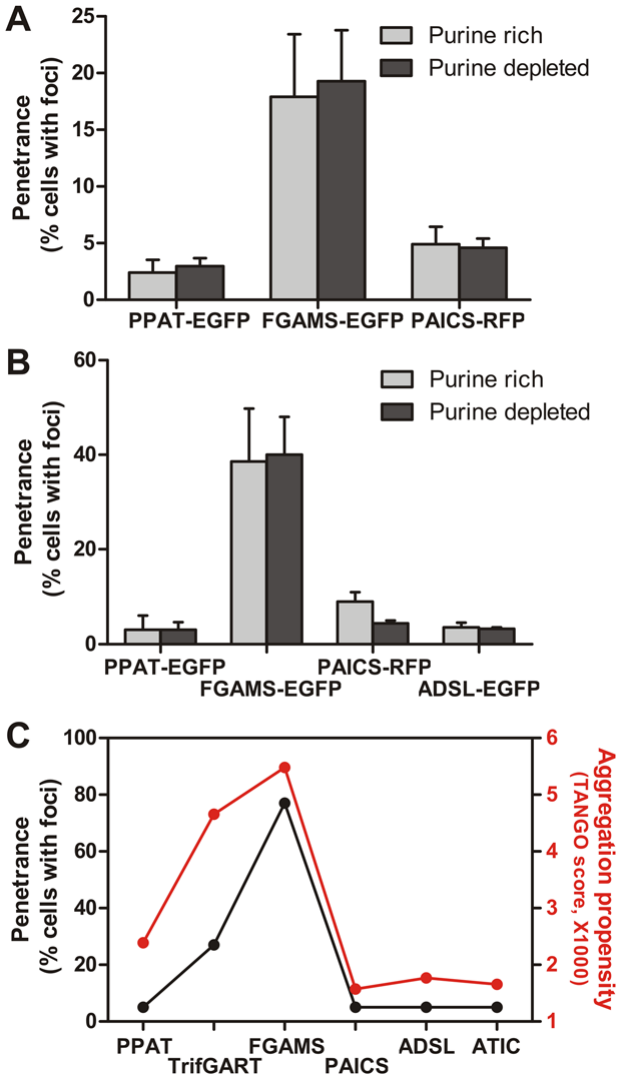
The formation of intracellular foci by transfected purine biosynthetic enzymes correlated well with the aggregation potential of the proteins but was not strongly influenced by purine availability. (**A**) The frequency with which intracellular bodies appeared across the population of transfected cells varied for individual *de novo* purine biosynthetic enzymes, and did not depend upon the purine content of the cell growth medium, shown here for HEK293 cells cultured using the optimized medium formulations as described in the Methods. Unless otherwise specified, for HEK293 cells assays in this and later figures, 200 ng of DNA were transfected for each construct. Bars in all experiments represent the average and +/- 1 s.d. from at least 3 replicates, counting *n*  =  769, 742, 755, 665, 1225, and 1016 cells, respectively. See Methods for abbreviations of protein names. (**B**) This trend was similar for HeLa cells cultured in purine-rich medium versus purine-depleted medium, formulated as in [6]. Unless otherwise specified, for HeLa cell assays in this and later figures, 1.6 µg of DNA were transfected for each construct. Bars in all experiments represent the average and +/- 1 s.d. from at least 3 replicates, here counting *n*  =  578, 821, 662, 832, 1519, 732, 674, and 791 total cells, respectively. (**C**) The frequency with which transfected cells exhibited bodies (shown here for values reported in [6]) correlated strongly with each protein’s predicted aggregation potential [10]. For comparison, the unrelated enzyme GAPDH has a TANGO score of 879.

We studied the mechanism by which transfected genes encoding human purine biosynthetic enzymes might be organized into cellular aggregates, especially under different nutrient conditions. HeLa and HEK293 cells transiently expressing GFP- or RFP-tagged purine biosynthetic enzymes were cultured in purine-poor media. Punctate bodies were observed, resembling those seen by An *et al*. [6]. While variable penetrance of different purine biosynthetic enzymes was confirmed **(** **Fig. 1A,B** **)**, we were surprised to find that this penetrance was largely independent of the presence or absence of purines in the growth medium (**Fig. 1A,B**).

We speculated that the formation of the punctate bodies might be due to factors other than purine starvation. Previously, the formation of yeast punctate bodies [5] was correlated with protein aggregation potentials (TANGO scores [10]), which suggested the possibility that the recombinant human proteins might be aggregating. Indeed, the fraction of cells in which individual purine biosynthetic enzymes formed detectable punctate bodies correlated well with the predicted aggregation potentials of the enzymes **(** **Fig. 1C** **)**.

**Figure 2:**
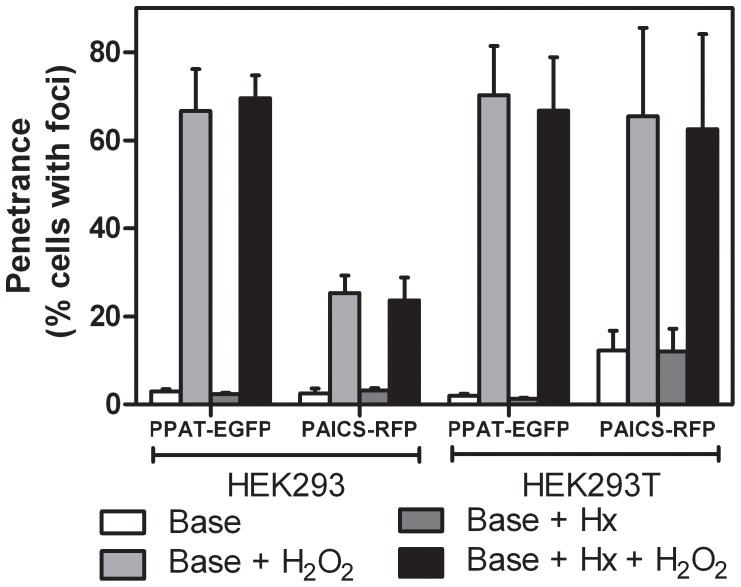
The cell stressor hydrogen peroxide (H_2_O_2_) strongly induced purine biosynthetic enzyme punctate bodies regardless of hypoxanthine (Hx) presence. Base medium is DMEM supplemented with 10% FBS. As indicated, medium was also supplemented with 1 mM H_2_O_2_ and/or 35 µM Hx as described in Methods. For HEK293 PPAT-EGFP cells, *n* = 4419, 2652, 3088, 3182 cells per bar. For HEK293 PAICS-RFP cells, *n* = 2970, 1944, 1880, 1760. For HEK293T PPAT-EGFP cells, *n* = 4537, 2267, 2411, 2947. For HEK293T PAICS-RFP cells, *n* = 4612, 3660, 4211, 3760. Bars indicate average +/- 1 s. d. across at least 3 replicates.

**Figure 3:**
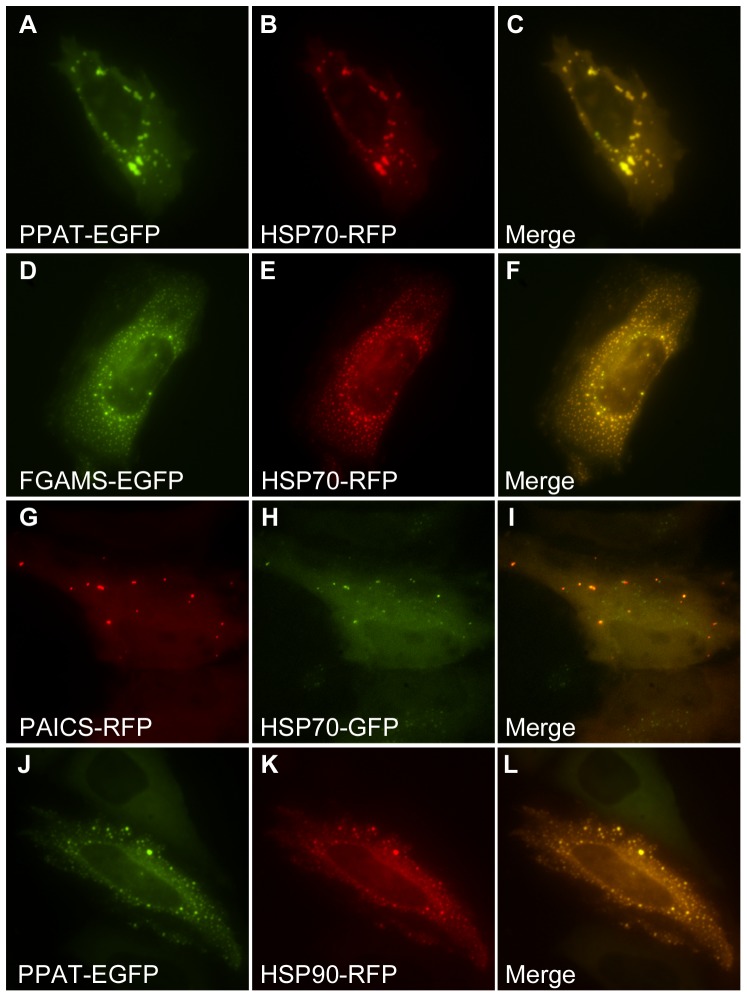
Co-expressed HSP70 and HSP90 chaperones marked purine biosynthesis bodies. Intracellular bodies formed by (**A**) PPAT-EGFP, (**D**) FGAMS-EGFP and (**G**) PAICS-RFP co-localized with co-transfected (**B,E**) HSP70-RFP or (**H**) HSP70-GFP, shown here in HeLa cells. (**C, F** and **I**) show merged images. (**J-L**) Intracellular bodies were also often observed to co-localize with co-transfected HSP90, shown here with PPAT-EGFP.

**Figure 4:**
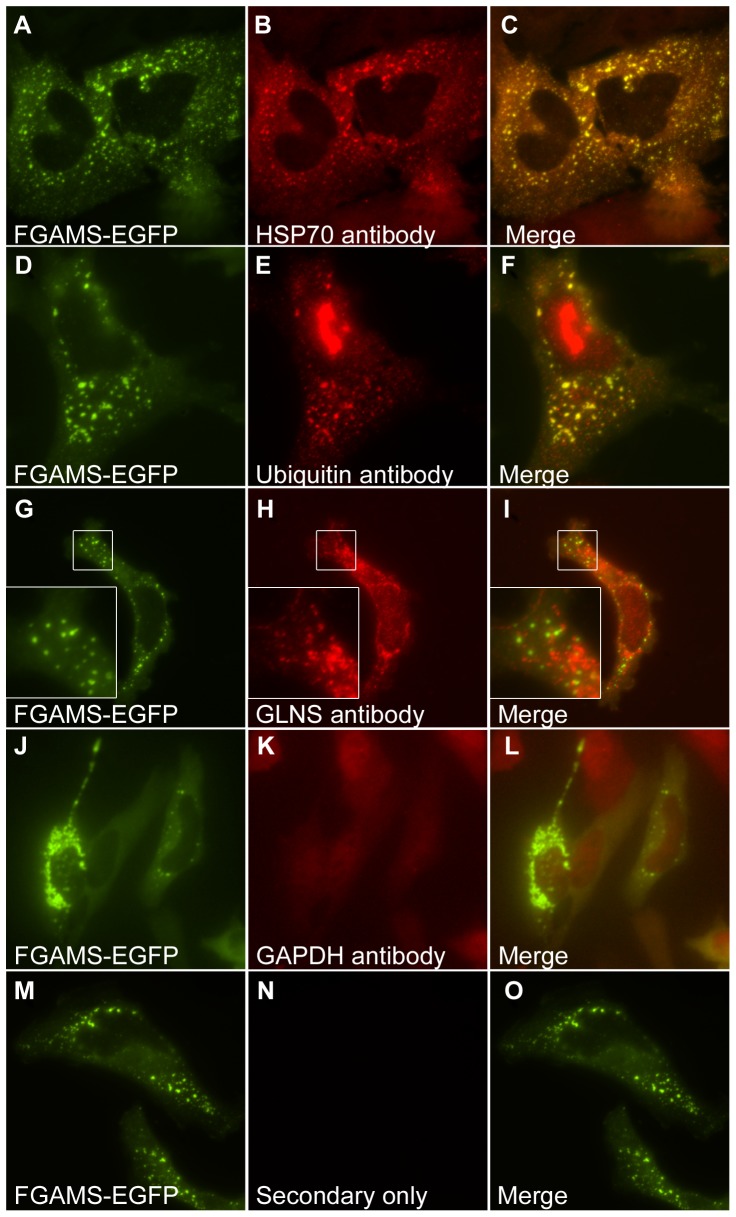
Endogenous markers of aggregated proteins associated with intracellular foci of transfected purine biosynthetic enzymes. (**A-C**) Endogenous HSP70 and (**D-F**) ubiquitin co-localized with bodies formed in cells transfected with FGAMS-EGFP, as assayed using immunofluorescence. Immunofluorescence against endogenous glutamine synthetase (**G-I**) or GAPDH (**J-L**), which are not markers for protein aggregation, confirmed that these proteins did not co-localize with the bodies. (**M-O**) Additional control experiments employing only the secondary antibodies (tested for both secondary antibodies and shown here for Alexa Fluor 594-conjugated goat anti-rabbit) exhibited no positive signal and, with the experiments in panels (**G-L**), ruled out the possibility of non-specific antibody-mediated localization to the bodies.

**Figure 5:**
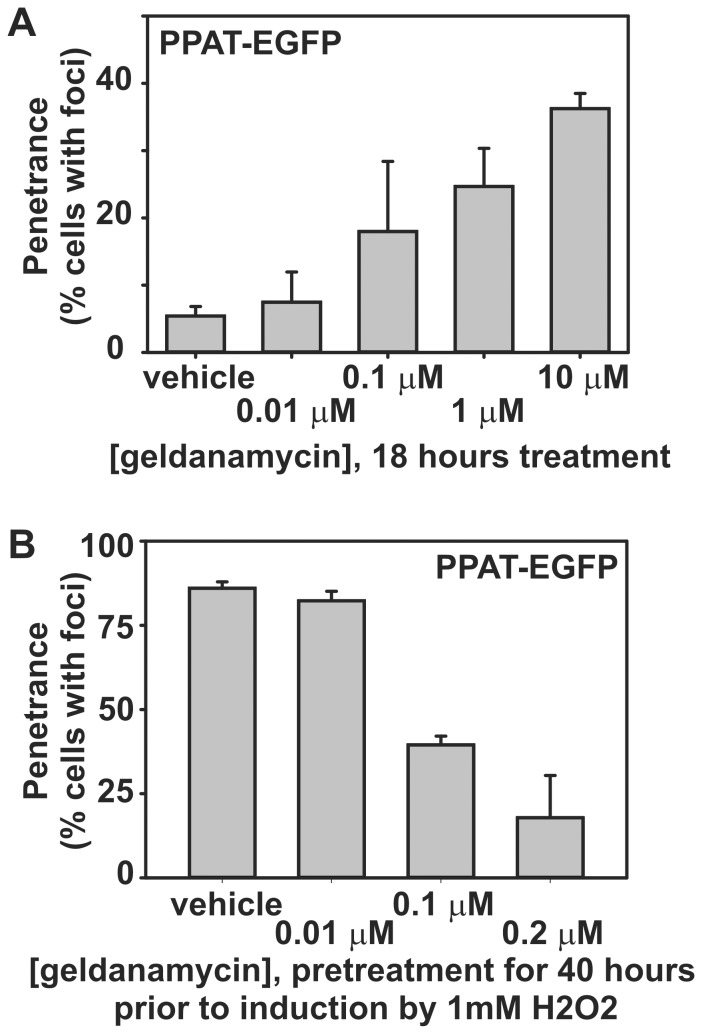
Chaperone activity modulated the formation of intracellular bodies of purine biosynthetic enzymes. (**A**) Short-term treatment with the HSP90 chaperone inhibitor geldanamycin induced puncta formation in a concentration-dependent manner, shown here for PPAT-EGFP in HEK293T cells. Bars indicate average +/- 1 s. d. across 3 replicates, *n*  =  680, 351, 565, 601, and 616 total cells, respectively. (**B**) Low-dosage geldanamycin pre-treatment—known to induce HSP70 activity [11]—suppressed oxidatively-induced puncta formation, shown here for PPAT-EGFP in HEK293T cells. Bars indicate average +/- 1 s. d. across 3 replicates, n  =  555, 500, 608, and 601 total cells, respectively. Results were similar for FGAMS-EGFP in the absence of hydrogen peroxide (data not shown).

If the proximal cause of the formation of punctate bodies was aggregation, then it seemed likely that: (a) such bodies could be induced by cellular stress, and (b) such bodies would contain proteins normally associated with aggregates, such as chaperones and ubiquitin. Indeed, we found that hydrogen peroxide can induce the formation of punctate bodies irrespective of the presence or absence of purines in the medium (**Fig. 2**). The punctate bodies appeared to be universally associated with the chaperone HSP70, as measured by co-transfection with a plasmid expressing fluorescently-tagged HSP70 (**Fig. 3A-I**). We similarly observed the chaperone HSP90 to co-localize to punctate bodies (**Fig. 3J-L**). We confirmed these results for endogenous HSP70, verifying using immunofluorescence that endogenous HSP70 localized with the punctate bodies (**Fig. 4A-C**). Association with HSP70 and HSP90 did not appear to be accidental; short-term treatment of cells with the HSP90 chaperone inhibitor geldanamycin increased accumulation of the punctate bodies (**Fig. 5A**). However, longer term treatment with low doses of geldanamycin is known to induce HSP70 chaperone production *via* hormesis [11], and accordingly longer term treatment with geldanamycin not only inhibited the formation of these bodies, but also prevented their induction by stressors, such as hydrogen peroxide **(** **Fig. 5B** **)**.

**Figure 6:**
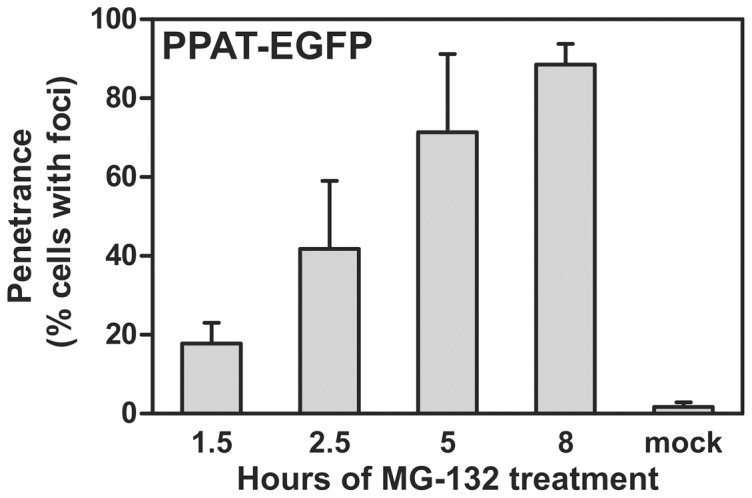
Inhibition of the proteasome with the drug MG-132 induced formation of PPAT-EGFP bodies in a time-dependent manner. HeLa cells treated with 20 µM MG-132 in DMSO for 1.5 hours, 2.5 hours, 5 hours, and 8 hours showed increasing fractions of the cell population exhibiting PPAT-EGFP puncta. Control treatments with only the carrier (DMSO) for 8 hours showed only minimal penetrance, consistent with no treatment (**Fig. 1B**). Bars indicate average +/- 1 s. d. across 3 replicates, *n*  =  625, 793, 1024, 466, and 397 cells, respectively.

As heat shock proteins are highly multifunctional and interact with many proteins in the cell, we further tested whether punctate bodies might be aggregates by assaying for their association with ubiquitin. Using immunofluorescence, we observed the co-localization of endogenous ubiquitin to the punctate bodies (**Fig. 4D-F**). In contrast, control experiments employing only secondary antibody or primary antibody targeting the unrelated enzymes GAPDH or glutamine synthetase showed no such co-localization with the punctate bodies (**Fig. 4G-O**). The association with ubiquitin suggested the possible involvement of the ubiquitin-proteasome proteolytic pathway; consistent with this involvement, we observed strong induction of punctate body formation by treatment with the proteasome inhibitor MG-132 (**Fig. 6**). The induction of punctate body formation by blocking protein degradation suggests that punctate bodies form in cases of excess protein buildup or compromised protein homeostasis, conditions which can lead to intracellular aggregation of proteins. (Interestingly, cell lines from purine salvage enzyme deficient mice are not hypersensitive to proteasome inhibitors [12].) Should the punctate bodies represent large protein aggregates, the bodies would likely be marked for degradation, consistent with their co-localization with ubiquitin. Thus, collectively, these data indicate that the punctate bodies associated strongly with known markers of aggregation, and that their formation is modulated in a manner consistent with aggregation [13], [14], [15], [16] .

**Figure 7:**
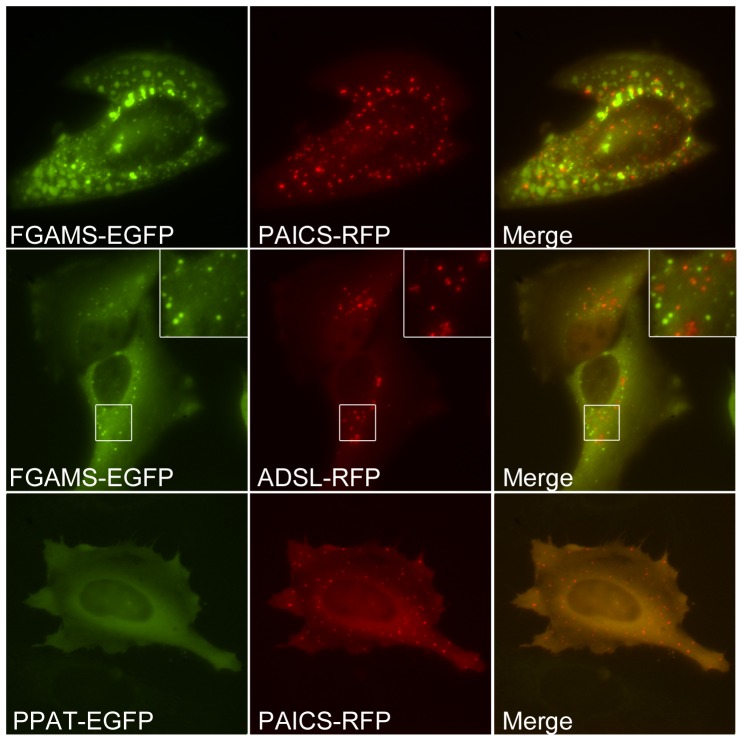
Purine biosynthesis enzymes only rarely co-localized in intracellular bodies, showing even partial co-localization in no more than 4% of co-transfected cells as assayed in HeLa cells and quantified in Table S1. The top row shows an example of partial but minimal co-localization of FGAMS-EGFP and PAICS-RFP bodies. The middle row shows an example of non-colocalizing FGAMS-EGFP and ADSL-RFP bodies. The bottom row shows an example of a more typical case, non-co-localization due to the formation of bodies by only one protein in doubly-transfected cells, as shown here for PPAT-EGFP and PAICS-RFP.

**Figure 8:**
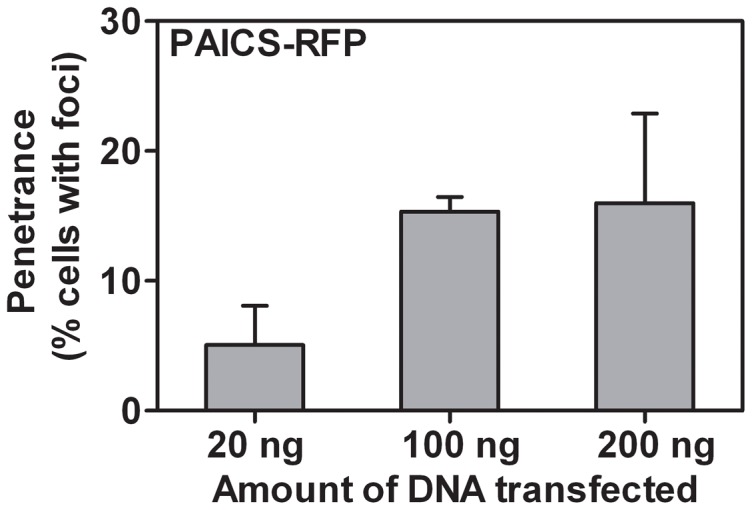
Formation of intracellular bodies in HEK293T cells scaled with DNA transfected. Among successfully transfected cells, the fraction of the cell population exhibiting PAICS-RFP puncta correlated strongly with the quantity of plasmid DNA transfected. Bars indicate average +/- 1 s. d. across at least 3 replicates, *n*  =  498, 627, and 591 cells, respectively.

If the observed bodies resulted from cellular stress and aggregation, then they might be the natural outcome of individual enzyme aggregation. If so, we would predict that other purine biosynthetic enzymes should not co-localize to the same protein puncta. Indeed, only ∼1% on average of co-transfected cells showed co-localization of different purine biosynthetic enzymes to the same puncta (**Fig. 7** **, Table S1**). As aggregation was proportional to the expression levels of individual enzymes (**Fig. 8**) and was stimulated by stress (**Fig. 2**), this limited co-localization likely derived from stress-induced co-aggregation. More generally, co-transfection tended to suppress puncta formation, or individual enzymes aggregated separately (**Table S1**). We also attempted to co-localize endogenous purine biosynthetic enzymes with protein puncta by use of immunofluorescence with antibodies against endogenous PPAT, PAICS, and TrifGART (data not shown). However, we did not find commercial antibodies satisfactory for immunofluorescence imaging, as all showed speckling in immunofluorescent experiments, irregardless of purine availability, and no co-localization with transfected recombinant proteins, arguing that the speckles are immunofluorescence artifacts.

**Figure 9:**
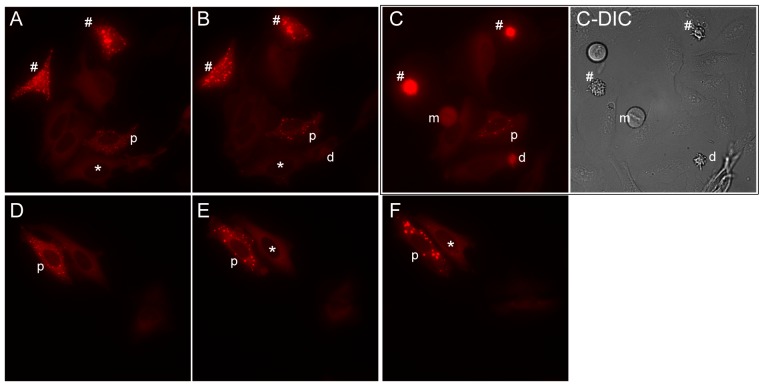
Time course imaging reveals that punctate bodies are dynamic, shown here for HeLa cells in purine-depleted medium transfected with PAICS-RFP grown in two replicate 4 hour time series. Panels (**A**) and (**D**) show the time zero condition; panels (**B**) and (**E**) show the same cells as in (**A**) and (**D**), respectively, following two hours of growth in the same medium; panels (**C**) and (**F**) show the same cells following two hours of growth after exchanging the growth medium to purine-rich. Cells marked by * display formation of punctate bodies over the time series, while cells marked by **p** display variable dynamics of punctate bodies. The # sign marks cells with punctate bodies that die over the course of the series; the cell marked by **d** dies in the absence of punctate bodies. Cell death was determined by marked cell shrinkage and membrane blebbing, detected by differential interference contrast (**DIC**) microscopy, as in panel (**C-DIC**), accompanied by markedly increased cellular fluorescence, easily distinguishable from flat healthy cells and mitotic cells (one is marked by **m** in panels (**C**) and (**C-DIC**)). Notably, punctate bodies are detectable in both purine-poor and rich media, with some forming even after the shift into purine-rich medium, as for the cell marked * in (**E-F**).

**Figure 10:**
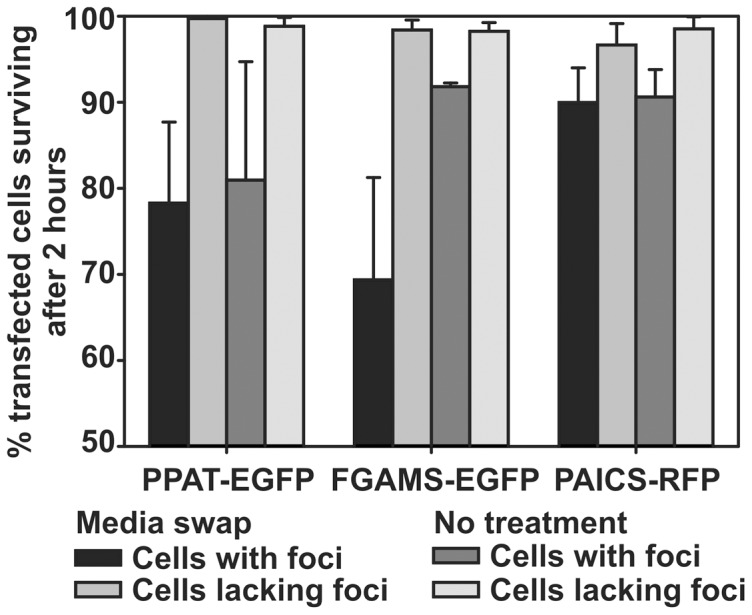
Transfected cells exhibiting intracellular bodies died at a significantly greater rate than transfected cells lacking such bodies, shown here for HeLa cells two hours after either no treatment or after exchanging the growth medium from purine-depleted to purine-rich. All comparisons between cells with and without intracellular bodies are statistically significant (*p*-values ranging from 10^-4^ to <10^-16^). Cell death was measured as marked cell shrinkage and membrane blebbing accompanied by markedly increased cellular fluorescence during time lapse fluorescence microscopy (e.g., as for the example cells in **Fig. 9**).

We saw additional support for the association between punctate bodies and cellular stress from time-lapse microscopy experiments of transfected cells, during which we observed variation both in punctate body dynamics as well as in cell survival (**Fig. 9**). Microscopy analysis of individual transfected cells over the course of 1-2 hours revealed that transfected cells without punctate bodies exhibited significantly higher survival rates than cells marked by punctate bodies (**Fig. 10**). This difference in survival rate was largely independent of purine availability, and persisted even when the cell growth medium was exchanged from purine-depleted to purine-rich. The observation that cells marked by punctate bodies died at greater rates than transfected cells lacking such aggregates suggested that these cells likely experienced greater levels of stress, although lacking in an established mechanism and known molecular signals, it is not clear from these data if the punctate bodies were a cause or consequence of that stress.

### Conclusions

These results serve as a cautionary tale for interpreting the cellular biology of transfected constructs. Our study encompassed transiently transfected cells expressing recombinant purine biosynthetic enzyme constructs, reproducing the conditions in which the original purinosome body was identified. We did not pursue the arduous task of generating stable cell lines. However, we point out that Goolijarsingh *et al.* [17] have tested a stably transfected TrifGART HEK293T cell line in which “the levels of GFP fusion protein produced within the cells are substantially reduced and are similar to wild-type levels” and found diffused signal throughout the cytoplasm and an absence of purinosome bodies. Although their cell growth condition of 7.7% FBS does not to lead easy direct comparison with our work or An *et al*.'s, it still supports our claim that transient transfection or otherwise out of context protein expression may play a role in generating punctate bodies.

In this regard, a consideration of the experiments of An *et al.* revealed that the purine-deficient medium did not merely lack purines, but differed in multiple other ways, as well (including the base medium, FBS concentration, and removal of nutrients up to 25 kDa), from the purine-rich medium, and the resulting cellular stress may have induced the aggregation of the recombinant proteins. A further indication that their purine-depleted medium may have been stress-inducing is their report that HTB-125 cells did not survive in their purine-depleted medium. These results also raise interesting questions regarding how growth conditions and cellular physiology impact the formation of single protein aggregates in general, especially proteins altered from their native state, for example whose levels may no longer correspond to normal, endogenous levels or which are expressed as fusion proteins. While it may be possible that purinosome bodies may shift to stress body-like protein complexes with increasing levels of protein expression or varying methods of cellular insults, we note that in general, the formation of purinosome bodies did not vary strongly amongst the different cell lines, and at least for PAICS-RFP, we observed the recombinant enzyme to be expressed within two-fold of endogenous levels **(Figure S1)**.

While it is possible that there are indeed endogenous purinosomes—notably, experiments by An and colleagues [6] and Baresova and colleagues [18] both present evidence for intracellular foci composed of endogenous purine biosynthetic enzymes as detected by immunofluorescence—these additional data suggest that the formation of punctate bodies in human cell culture following transient transfection of clones expressing purine biosynthesis enzymes can possibly be explained as protein aggregation.

## Materials and Methods

### Abbreviations of protein names

**PPAT**, phosphoribosyl pyrophosphate amidotransferase; **TrifGART**, the trifunctional enzyme glycinamide ribonucleotide (GAR) synthetase, GAR transformylase, and aminoimidazole ribonucleotide synthetase; **FGAMS**, formylglycinamidine ribonucleotide synthase; **PAICS**, the bifunctional enzyme carboxyaminoimidazole ribonucleotide synthase and succinylaminoimidazolecarboxamide ribonucleotide synthetase; **ADSL**, adenylosuccinate lyase; **ATIC**, the bifunctional enzyme aminoimidazolecarboxamide ribonucleotide transformylase and IMP cyclohydrolase; **GFP**, green fluorescent protein; **RFP**, red fluorescent protein; **EFGP**, enhanced GFP; **GAPDH**, glyceraldehyde 3-phosphate dehydrogenase; **GLNS**, glutamine synthetase.

### Expression Vectors

The PPAT-EGFP and FGAMS-EGFP expression plasmids were generously provided by An and Benkovic [6]. The ADSL-EGFP expression plasmid was constructed by Gateway cloning into the FGAMS-EGFP expression plasmid, modified to introduce the Invitrogen pDEST47 Gateway cassette in place of FGAMS. All other cDNAs were obtained from the human ORFeome collection (OpenBiosystems) and cloned using Gateway cloning into either the pcDNA-DEST47 plasmid (Invitrogen) for carboxy-terminal GFP-tagged expression clones or the pTagRFP-N plasmid (Evrogen; modified to introduce the Invitrogen pDEST47 Gateway cassette) for carboxy-terminal RFP-tagged expression clones.

### Cell Culture

To replicate conditions reported by An *et al*. to give rise to the purinosome bodies, HeLa cells obtained from the American Type Culture Collection (ATCC) were cultured at 37°C with 5% CO_2_ in the following medium as previously described [6]: Purine rich medium: MEM medium supplemented with 10% FBS (fetal bovine serum) and 50 µg/mL gentamycin sulfate (Invitrogen); purine-depleted medium: RPMI medium supplemented with 5% dialyzed FBS and 50 µg/mL gentamycin. FBS was dialyzed against 100-fold volumes of 0.9% NaCl at 4 °C for 2-4 days using a 25 kDa molecular weight cut-off (MWCO) dialysis membrane with daily exchange of the dialysis solution.

As a test of alternate cell lines and matched purine-rich and purine-depleted media formulations, HEK293 or HEK293T cells obtained from ATCC were cultured at 37°C with 5% CO_2_ in the following media: (1) Optimized purine-rich medium: DMEM medium supplemented with 10% FBS and 35 µM hypoxanthine, or (2) Optimized purine-depleted medium: DMEM medium supplemented with 10% dialyzed FBS. To remove purines but retain other important factors, FBS was dialyzed against 100-fold volumes of 0.9% NaCl at 4°C for 2-4 days using a 1 kDa MWCO dialysis membrane with daily exchange of the dialysis solution [19], [20].

One day prior to transfection, HeLa cells were plated in 6-well glass bottom plates in either purine-rich or purine-depleted medium lacking antibiotics. HEK293 or HEK293T cells were plated in 96-well glass bottom plates in the optimized purine-rich medium lacking antibiotics. Plasmids were transfected into cells using Lipofectamine 2000 (Invitrogen) and Opti-MEM reduced serum medium (Invitrogen) according to the manufacturer’s instructions. The transfection medium was replaced with fresh purine-rich or purine-depleted medium 5 hours after transfection. Immediately before imaging, cells were washed once with their respective growth medium. Cells were imaged in their respective growth medium to minimize stress imposed by nutrient shifts. We note that the imaging medium therefore contained phenol red, which could potentially reduce the signal-to-noise ratio, but in practice, we did not experience problems in detecting and visualizing purinosome bodies. Live cells were imaged ∼20-24 hours after transfection.

For MG-132 experiments, HeLa cells were plated in 96-well glass bottom plates in DMEM medium supplemented with 10% FBS. Due to the toxic nature of MG-132, we opted to use a less toxic transfection reagent than Lipofectamine 2000, and therefore, we transfected the PPAT-EGFP-expressing plasmid into cells using Fugene HD (Roche Applied Sciences) and Opti-MEM reduced serum medium (Invitrogen) using a 2:4.5 DNA to transfection reagent ratio following the manufacturer's protocol. The transfection medium was replaced with fresh DMEM+10% FBS about 5 hours following transfection.

### Immunofluorescence

For immunofluorescence experiments, cells were fixed with 3.7% methanol-free formaldehyde freshly diluted from 16% stock (28908, Thermo Scientific) at 37°C for 15-20 minutes, blocked with 5% goat serum in PBS-T buffer for 30-60 minutes at room temperature, then incubated with primary antibody overnight at 4°C. Cells were washed with PBS buffer, then incubated with secondary antibody for 1 hour. Primary antibodies used: HSP70 (ab5439, Abcam), ubiquitin (ab7780, Abcam), GAPDH (sc-32233, Santa Cruz Biotech.), glutamine synthetase (sc-9067, Santa Cruz Biotech.). Secondary antibodies used: Alexa Fluor 594-conjugated goat anti-mouse (Invitrogen), Alexa Fluor 594-conjugated goat anti-rabbit (Invitrogen). All antibodies were used at the manufacturer’s recommended concentrations. We additionally tested the protocol using 2% methanol-free formaldehyde fixation or 2% goat serum in PBS-T block at the appropriate steps to find no appreciable differences.

Tested antibodies that proved unsuitable or inconclusive for immunofluorescence included the anti-PPAT antibodies sc-101892 (Santa Cruz Biotech.) and ab71340 (Abcam), the anti-PAICS antibody sc-16150 (Santa Cruz Biotech.), and the anti-TrifGART antibody H00002618-M01 (Novus Biologicals).

### Western Blot

For western blotting, cells were grown in DMEM media containing 10% FBS and transfected as described above with 1.6 µg or 200 ng DNA (HeLa or HEK cells, respectively), in regular 96-well plates (HEK293 and HEK293T) or 6-well plates (HeLa). Cells were washed with cold PBS buffer then extracted with Laemmaeli sample buffer containing 5% beta-mercaptoethanol. Total protein was separated on a 4–15% Mini-PROTEAN TGX precast gel (BioRad) and blotted with PVDF membrane. Primary antibodies used: anti-PAICS (HPA035895, Sigma) and anti-GAPDH (sc-32233, Santa Cruz Biotech.). Secondary antibodies: goat anti-rabbit IgG F(ab')_2_-HRP (sc-3837, Santa Cruz Biotech.) and goat anti-mouse IgG F(ab')_2_-HRP (sc-3697, Santa Cruz Biotech.). Membranes were scanned with an ImageQuant LAS 4000 (GE Healthcare).

### Fluorescent Microscopy of Cells

Live cells were imaged using a Nikon Eclipse TE2000-E inverted microscope inside a chamber maintained at 37°C and 5% CO_2_. Fixed cells were imaged at room temperature without CO_2_ supplement. Images were acquired using a Photometrics Cascade II 512 camera and Nikon Plan Apo 40x/0.95, 60x/0.95, 60x/1.40, or 100x/1.40 objectives. GFP detection was accomplished using a ET490/20x excitation filter (Chroma Technology), ET525/36 m emission filter (Chroma Technology) and 89100 bs dichroic (Chroma Technology), and RFP detection was carried out using a ET555/25x excitation filter (Chroma Technology), ET605/52 m emission filter (Chroma Technology) and 89100 bs dichroic (Chroma Technology). The same excitation and emission conditions were used for each GFP variant. We did not detect notable fluorescence emission bleedthrough or photobleaching with these settings. Filter wheels (Sutter Lambda 10-3), motorized stage (Prior H117), and image acquisition were driven by NIS Elements AR Imaging software.

### Cell Counts

Cell counts measuring penetrance of punctate bodies reflect apparently healthy adherent cells only. Mitotic cells (identified as rounded cells with a visible rod-like central structure spanning the cell’s DIC image accompanied by a corresponding decrease of fluorescent signal; see cell **m** in **Fig. 9C and C** **-DIC** for an example) and dead cells were not counted for measuring penetrance of punctate body formation. (For the survival rate analyses only, dead HeLa cells were selected based on dramatic cell shrinkage, membrane blebbing, and intense fluorescence, e.g. as seen for the cells marked by **#** in **Fig. 9**. We note that it is nonetheless possible that only a subset of cell death events—those accompanied by marked visible cellular reorganization—are counted by this assay.) Cells displaying any morphology of punctate bodies were counted, regardless of number of punctate bodies per cell or ranging sizes of those punctate bodies. We observed a spectrum of morphologies which were dynamic; for example, over the course of unperturbed growth, we observed both increases and decreases in the numbers of punctate bodies per cell. Future work may address and characterize the different morphologies and kinetics of punctate bodies.

### Drug Treatments

For all drug treatments, drugs were diluted to their final concentrations in prewarmed cell growth medium then added to cells for the duration of the treatment. Hydrogen peroxide was added to cells at a concentration of 1 mM for 0.5-1.5 hours before imaging. For short-term treatment, geldanamycin was added to cells at the indicated concentrations at the time of post-transfection medium replacement, and the cells were incubated for 18 hours. For long-term treatment, geldanamycin was added to cells at the indicated concentrations 16 hours prior to transfection, for a total incubation time of 40 hours. MG-132 was added to cells at a final concentration of 20 µM for the indicated time intervals. Cells were fixed at each MG-132 time point with 3.7% formaldehyde for 15-20 minutes and washed 3 times with PBS before imaging.

## Supporting Information

Figure S1 **Representative expression levels of PAICS-RFP transfected constructs in comparison to the endogenous PAICS protein.** HeLa (**A,D**), HEK293 (**B,E**) and HEK293T (**C,F**), untransfected (U) or transfected with PAICS-RFP (T) under representative growth conditions are probed with anti-PAICS antibody. (**D-F**) Representative variation of construct expression between independent transfections. Quantified expression level ratios of PAICS-RFP construct to endogenous PAICS (**G**) across all cells, adjusted with respective transfection rates (ranging from 6-32%) measured by microscopy, show comparable expression (within two-fold) of the construct and endogenous enzyme in transfected cells, under conditions (see Methods) in which PAICS-RFP foci are observed. Expression ratios are calculated as (PAICS-RFP Western blot intensity)/(endogenous PAICS Western blot intensity * transfection efficiency). Bars indicate average +/- 1 s. d. across at least 3 replicates. (**H**) Representative HEK293 cells corresponding to the bar in (**G**) and showing visible foci. (**I**) Expression levels of PAICS-RFP are not strongly altered by hydrogen peroxide and/or hypoxanthine treatment for HEK293 (lanes 2-5) and HEK293T cells (lanes 1, 6-9).
(TIF)

Table S1 **Co-transfection significantly suppresses formation of intracellular punctate bodies, arguing against the bodies representing active purinosomes, as quantified for different purine biosynthetic enzyme pairs, reporting counts and percentages of successfully co-transected HeLa cells as a function of protein localization.**
(XLS)

## References

[1] Brengues M, Teixeira D, Parker R (2005) Movement of eukaryotic mRNAs between polysomes and cytoplasmic processing bodies. Science 310: 486–489. 16141371 10.1126/science.1115791 PMC1863069

[2] Sagot I, Pinson B, Salin B, Daignan-Fornier B (2006) Actin bodies in yeast quiescent cells: an immediately available actin reserve? Mol Biol Cell 17: 4645–4655. 16914523 10.1091/mbc.E06-04-0282 PMC1635378

[3] Alberti S, Halfmann R, King O, Kapila A, Lindquist S (2009) A systematic survey identifies prions and illuminates sequence features of prionogenic proteins. Cell 137: 146–158. 19345193 10.1016/j.cell.2009.02.044 PMC2683788

[4] Johnston JA, Ward CL, Kopito RR (1998) Aggresomes: a cellular response to misfolded proteins. J Cell Biol 143: 1883–1898. 9864362 10.1083/jcb.143.7.1883 PMC2175217

[5] Narayanaswamy R, Levy M, Tsechansky M, Stovall GM, O'Connell JD, et al (2009) Widespread reorganization of metabolic enzymes into reversible assemblies upon nutrient starvation. Proc Natl Acad Sci U S A 106: 10147–10152. 19502427 10.1073/pnas.0812771106 PMC2691686

[6] An S, Kumar R, Sheets ED, Benkovic SJ (2008) Reversible compartmentalization of de novo purine biosynthetic complexes in living cells. Science 320: 103–106. 18388293 10.1126/science.1152241

[7] An S, Deng Y, Tomsho JW, Kyoung M, Benkovic SJ (2010) Microtubule-assisted mechanism for functional metabolic macromolecular complex formation. Proc Natl Acad Sci U S A 107: 12872–12876. 20615962 10.1073/pnas.1008451107 PMC2919939

[8] An S, Kyoung M, Allen JJ, Shokat KM, Benkovic SJ (2010) Dynamic regulation of a metabolic multi-enzyme complex by protein kinase CK2. J Biol Chem 285: 11093–11099. 20157113 10.1074/jbc.M110.101139 PMC2856985

[9] Verrier F, An S, Ferrie AM, Sun H, Kyoung M, et al (2011) GPCRs regulate the assembly of a multienzyme complex for purine biosynthesis. Nat Chem Biol 7: 909–915. 22020552 10.1038/nchembio.690 PMC3218230

[10] Fernandez-Escamilla AM, Rousseau F, Schymkowitz J, Serrano L (2004) Prediction of sequence-dependent and mutational effects on the aggregation of peptides and proteins. Nat Biotechnol 22: 1302–1306. 15361882 10.1038/nbt1012

[11] McLean PJ, Klucken J, Shin Y, Hyman BT (2004) Geldanamycin induces Hsp70 and prevents alpha-synuclein aggregation and toxicity in vitro. Biochem Biophys Res Commun 321: 665–669. 15358157 10.1016/j.bbrc.2004.07.021

[12] Lewers JC, Ceballos-Picot I, Shirley TL, Mockel L, Egami K, et al (2008) Consequences of impaired purine recycling in dopaminergic neurons. Neuroscience 152: 761–772. 18313225 10.1016/j.neuroscience.2007.10.065 PMC3498629

[13] Howard RA, Sharma P, Hajjar C, Caldwell KA, Caldwell GA, et al (2007) Ubiquitin conjugating enzymes participate in polyglutamine protein aggregation. BMC Cell Biol 8: 32. 17663792 10.1186/1471-2121-8-32 PMC1952058

[14] Sampathu DM, Giasson BI, Pawlyk AC, Trojanowski JQ, Lee VM (2003) Ubiquitination of alpha-synuclein is not required for formation of pathological inclusions in alpha-synucleinopathies. Am J Pathol 163: 91–100. 12819014 10.1016/s0002-9440(10)63633-4 PMC1868149

[15] Kim S, Nollen EA, Kitagawa K, Bindokas VP, Morimoto RI (2002) Polyglutamine protein aggregates are dynamic. Nat Cell Biol 4: 826–831. 12360295 10.1038/ncb863

[16] Proctor CJ, Lorimer IA (2011) Modelling the role of the Hsp70/Hsp90 system in the maintenance of protein homeostasis. PLoS One 6: e22038. 21779370 10.1371/journal.pone.0022038 PMC3137010

[17] Gooljarsingh LT, Ramcharan J, Gilroy S, Benkovic SJ (2001) Localization of GAR transformylase in Escherichia coli and mammalian cells. Proc Natl Acad Sci U S A 98: 6565–6570. 11381136 10.1073/pnas.121182998 PMC34393

[18] Baresova V, Skopova V, Sikora J, Patterson D, Sovova J, et al (2012) Mutations of ATIC and ADSL affect purinosome assembly in cultured skin fibroblasts from patients with AICA-ribosiduria and ADSL deficiency. Hum Mol Genet 21: 1534–1543. 22180458 10.1093/hmg/ddr591

[19] Yamaoka T, Kondo M, Honda S, Iwahana H, Moritani M, et al (1997) Amidophosphoribosyltransferase limits the rate of cell growth-linked de novo purine biosynthesis in the presence of constant capacity of salvage purine biosynthesis. J Biol Chem 272: 17719–17725. 9211923 10.1074/jbc.272.28.17719

[20] Yamaoka T, Yano M, Kondo M, Sasaki H, Hino S, et al (2001) Feedback inhibition of amidophosphoribosyltransferase regulates the rate of cell growth via purine nucleotide, DNA, and protein syntheses. J Biol Chem 276: 21285–21291. 11290738 10.1074/jbc.M011103200

